# Refractiveindex.info database of optical constants

**DOI:** 10.1038/s41597-023-02898-2

**Published:** 2024-01-18

**Authors:** Mikhail N. Polyanskiy

**Affiliations:** Brookhaven National Laboratory, Accelerator Test Facility, Upton, NY 11973 USA

**Keywords:** Optical materials and structures, Materials for optics

## Abstract

We introduce the *refractiveindex.info* database, a comprehensive open-source repository containing optical constants for a wide array of materials, and describe in detail the underlying dataset. This collection, derived from a meticulous compilation of data sourced from peer-reviewed publications, manufacturers’ datasheets, and authoritative texts, aims to advance research in optics and photonics. The data is stored using a YAML-based format, ensuring integrity, consistency, and ease of access. Each record is accompanied by detailed metadata, facilitating a comprehensive understanding and efficient utilization of the data. In this descriptor, we outline the data curation protocols and the file format used for data records, and briefly demonstrate how the data can be organized in a user-friendly fashion akin to the books in a traditional library.

## Background & summary

The complex index of refraction $\widetilde{n}=n+ik$ is essential for understanding the optical properties of materials due to its close relation with the relative permittivity *ε*_*r*_ (${\varepsilon }_{r}={\widetilde{n}}^{2}$ in the case of non-magnetic materials^1^). In this context, *n* is the phase velocity ratio of light in a vacuum to that in the material, representing refraction. The extinction coefficient *k* measures absorption. The absorption coefficient *α* can be expressed as $\alpha =4\pi k/\lambda$, with *λ* representing the light’s wavelength.

Accurately determining both *n* and *k*, and their chromatic dispersion, is crucial in science, engineering, and 3D artistic rendering. The intricate design of optical instruments, particularly those minimizing aberrations, depends heavily on understanding the *n*(*λ*) function of transparent optical materials^2^. Cross-disciplinary efforts to characterize optical material properties have resulted in a wealth of datasets and analytical formulas, enhancing our grasp and utilization of optical constants.

Initiatives to amalgamate scattered information on optical constants resulted in seminal works like Palik’s comprehensive compilation^3^, offering structured and uniformly formatted data. Though monumental, the rapid advancement of technology and research methodologies signposted the need for a more dynamic, easily accessible repository of *n* and *k* values. This need intensified with the emergence of high-peak-power lasers, highlighting the critical role of the nonlinear refractive index, *n*_2_, in decoding material behavior under intense light. *n*_2_ characterizes the refractive index’s variation with the optical field intensity *I*, expressed as *n* = *n*_0_ + *n*_2_*I*, where *n*_0_ is the refractive index at zero intensity.

The *refractiveindex.info* database emerged in response, offering a systematically organized, dynamic repository of optical constants. Since its inception in 2008, continuous enhancements have established it as a reliable resource, with the YAML-based file format ensuring data integrity and ease of access. The integration of the *n*_2_ database in 2023 reaffirms our dedication to addressing modern challenges in optics and photonics.

The subsequent sections delve into the methods employed for collecting the data included in *refractiveindex.info*, detailing the file format used for storing data records and our approach to verifying dataset integrity. We then highlight the application aspects of this dataset, illustrated with an example of an interactive data browser, and conclude with a statement on data availability.

## Methods

### Data source clarification

It is pivotal to underscore that our work does not generate original experimental data. Instead, we create a comprehensive data repository, systematizing and cataloging existing optical constants published by others. This approach ensures a diverse and rich collection of verified data is readily accessible for various applications in a standardized format. As a result, this section does not describe experimental procedures but focuses on the methodologies employed in data collection and formatting.

### Data collection

Data on the optical properties of materials is aggregated from a variety of publicly accessible and credible sources, ensuring a wide range of optical constants is represented. The systematic collection process involves categorizing the data based on the type of material, its properties, and the source of information. The entire process is mostly manual due to the large variety of data sources and presentation methods. Below, we detail the steps typically undertaken to identify sources, extract data, and convert it into a unified format used in the data records.

1. Identification of data sources

- Existing data collections: The initial sources for literature containing data on optical constants were published collections, such as those included in the Handbook of Optics^4^. The publications referenced therein constituted our initial list of data sources.
- Cross-references from the initial list of data sources: In the next iteration, we scan for references to other publications in the sources identified in the previous step (usually, scientific papers reporting new measurements describe previous works on similar topics).
- Search engines: We perform a broader search for publications on optical properties of materials using tools like Google Scholar to find newer references and those that may have been missed in earlier steps.
- Glass makers’ catalogs: We checked the websites of glass manufacturers for catalogs of glasses they produce. These catalogs are often available in Zemax AGF format, suitable for automatic data extraction.
- User input: At the present maturity level of the project, most new data sources are recommended by users. Researchers often provide us with data in a format ready-to-include in the *refractiveindex.info* dataset as soon as the data are first published.

2. Data extraction

- Dispersion formula: When the linear refractive index as a function of wavelength is given as a dispersion formula, the coefficients are manually transferred to the data record. Adjustments are made if necessary to fit the formula to one of the standard forms documented in the following section.
- Tabulated numerical data: In cases where optical constants are presented in tabulated numerical form, the data are transferred to the data record with minimal changes required by the used format (e.g., using micrometer as the unit of wavelength and expressing internal absorption by extinction coefficient *k*). This process is simplified if data is in a standard table format (e.g., CSV or Microsoft Excel) in supplementary materials or directly provided by authors. For older publications, typically available as bitmap-based PDF files, text recognition options in PDF reading software prove helpful.
- Models: If a reference presents a model for calculating optical constants, a Python script is developed to generate a tabulated data record compatible with the required format. This script is then made publicly available to *refractiveindex.info* users on GitHub.
- Graphical-only data: Occasionally, authors choose to include only graphical data in publications without numerical data. In such cases, we first attempt to contact the authors for the original data. If this is not feasible (in the case of older publications) or if there is no response, semi-automatic digitization of the plots is sometimes performed (e.g., using Engauge Digitizer software); however, this is considered a last resort due to its time-consuming and less accurate nature and is typically used only when no alternative data is available.
- Glass catalogs: Glass makers’ catalogs in AGF format are automatically converted into *refractiveindex.info* data records using a dedicated Python script.

### Data formatting and updates

All collected data are converted into a standardized format stored in human-readable YAML files, as detailed in the following section. This approach ensures consistency and facilitates easy access and manipulation by both users and computer programs.

Errors in converting original data to standardized data records occasionally occur and are typically reported by users. All efforts are made to implement necessary corrections promptly via regular updates of the dataset.

## Data Records

The dataset is available at *Figshare*^5^.

### YAML-based file format for data records

The data records employ YAML-based file format (https://yaml.org), ensuring the data is both easily readable and maintainable. Each YAML file encompasses data related to a specific material, evaluated under defined conditions and reported in a particular publication, with a primary focus on the refractive index and extinction coefficient.

As an illustrative example, consider a data record for SiO_2_ in which the refractive index *n* is expressed through a dispersion formula. The associated YAML file is located at data-nk/main/SiO2/Malitson.yml and is organized as follows:


REFERENCES: “1) I. H. Malitson, J. Opt. Soc. Am. 55, 1205-1209 (1965)...


COMMENTS: “Fused silica, 20 °C”DATA:– type: formula 1wavelength_range: 0.21 6.7coefficients: 0 0.6961663 0.0684043 0.4079426...SPECS:n_is_absolute: falsewavelength_is_vacuum: false

temperature: 20 °C

Every YAML data file primarily consists of two mandatory fields: REFERENCES and DATA. REFERENCES cite the source of the data, while DATA provides the values of the optical constants. There are also two optional fields, COMMENTS and SPECS, offering additional context and structured information respectively.

#### DATA

In the case of a data record for linear optical constants, the DATA field can be specified as a dispersion formula, identified by a formula number, or as tabulated data sets of *n*(*λ*) and/or *k*(*λ*). For dispersion formulas, the ‘wavelength_range’ entry indicates the applicable wavelength range for the data, always expressed in micrometers. Each dispersion formula in the database is numerically identified and described as follows:

1 $${n}^{2}-1={C}_{1}+\frac{{C}_{2}{\lambda }^{2}}{{\lambda }^{2}-{C}_{3}^{2}}+\frac{{C}_{4}{\lambda }^{2}}{{\lambda }^{2}-{C}_{5}^{2}}+\frac{{C}_{6}{\lambda }^{2}}{{\lambda }^{2}-{C}_{7}^{2}}+\frac{{C}_{8}{\lambda }^{2}}{{\lambda }^{2}-{C}_{9}^{2}}+\frac{{C}_{10}{\lambda }^{2}}{{\lambda }^{2}-{C}_{11}^{2}}+\frac{{C}_{12}{\lambda }^{2}}{{\lambda }^{2}-{C}_{13}^{2}}+\frac{{C}_{14}{\lambda }^{2}}{{\lambda }^{2}-{C}_{15}^{2}}+\frac{{C}_{16}{\lambda }^{2}}{{\lambda }^{2}-{C}_{17}^{2}}$$

2 $${n}^{2}-1={C}_{1}+\frac{{C}_{2}{\lambda }^{2}}{{\lambda }^{2}-{C}_{3}}+\frac{{C}_{4}{\lambda }^{2}}{{\lambda }^{2}-{C}_{5}}+\frac{{C}_{6}{\lambda }^{2}}{{\lambda }^{2}-{C}_{7}}+\frac{{C}_{8}{\lambda }^{2}}{{\lambda }^{2}-{C}_{9}}+\frac{{C}_{10}{\lambda }^{2}}{{\lambda }^{2}-{C}_{11}}+\frac{{C}_{12}{\lambda }^{2}}{{\lambda }^{2}-{C}_{13}}+\frac{{C}_{14}{\lambda }^{2}}{{\lambda }^{2}-{C}_{15}}+\frac{{C}_{16}{\lambda }^{2}}{{\lambda }^{2}-{C}_{17}}$$

3 $${n}^{2}={C}_{1}+{C}_{2}{\lambda }^{{C}_{3}}+{C}_{4}{\lambda }^{{C}_{5}}+{C}_{6}{\lambda }^{{C}_{7}}+{C}_{8}{\lambda }^{{C}_{9}}+{C}_{10}{\lambda }^{{C}_{11}}+{C}_{12}{\lambda }^{{C}_{13}}+{C}_{14}{\lambda }^{{C}_{15}}+{C}_{16}{\lambda }^{{C}_{17}}$$

4 $${n}^{2}={C}_{1}+\frac{{C}_{2}{\lambda }^{{C}_{3}}}{{\lambda }^{2}-{C}_{4}^{{C}_{5}}}+\frac{{C}_{6}{\lambda }^{{C}_{7}}}{{\lambda }^{2}-{C}_{8}^{{C}_{9}}}+{C}_{10}{\lambda }^{{C}_{11}}+{C}_{12}{\lambda }^{{C}_{13}}+{C}_{14}{\lambda }^{{C}_{15}}+{C}_{16}{\lambda }^{{C}_{17}}$$

5 $$n={C}_{1}+{C}_{2}{\lambda }^{{C}_{3}}+{C}_{4}{\lambda }^{{C}_{5}}+{C}_{6}{\lambda }^{{C}_{7}}+{C}_{8}{\lambda }^{{C}_{9}}+{C}_{10}{\lambda }^{{C}_{11}}$$

6 $$n-1={C}_{1}+\frac{{C}_{2}}{{C}_{3}-{\lambda }^{-2}}+\frac{{C}_{4}}{{C}_{5}-{\lambda }^{-2}}+\frac{{C}_{6}}{{C}_{7}-{\lambda }^{-2}}+\frac{{C}_{8}}{{C}_{9}-{\lambda }^{-2}}+\frac{{C}_{10}}{{C}_{11}-{\lambda }^{-2}}$$

7 $$n={C}_{1}+\frac{{C}_{2}}{{\lambda }^{2}-0.028}+{C}_{3}{\left(\frac{1}{{\lambda }^{2}-0.028}\right)}^{2}+{C}_{4}{\lambda }^{2}+{C}_{5}{\lambda }^{4}+{C}_{6}{\lambda }^{6}$$

8 $$\frac{{n}^{2}-1}{{n}^{2}+2}={C}_{1}+\frac{{C}_{2}{\lambda }^{2}}{{\lambda }^{2}-{C}_{3}}+{C}_{4}{\lambda }^{2}$$

9 $${n}^{2}={C}_{1}+\frac{{C}_{2}}{{\lambda }^{2}-{C}_{3}}+\frac{{C}_{4}(\lambda -{C}_{5})}{{(\lambda -{C}_{5})}^{2}+{C}_{6}}$$

In another example, found at data-nk/main/Si/Aspnes.yml, *n*(*λ*) and *k*(*λ*) for Silicon (Si) are provided in tabulated numerical form:


DATA:- type: tabulated nkdata: |0.2066 1.010 2.9090.2101 1.083 2.982...


Here, the first column corresponds to the wavelength in micrometers, while the others represent the unitless refractive index *n* and extinction coefficient *k*. Alternatively, *n* and *k* can be outlined separately in two two-column entries. A data entry might also combine a dispersion formula for *n* with numerical data for *k*, as illustrated in the SCHOTT N-BK7 glass dataset at data-nk/glass/schott/N-BK7.yml:


DATA:- type: formula 2wavelength_range: 0.3 2.5coefficients: 0 1.03961212 0.00600069867 0.231792344...- type: tabulated kdata: |0.300 2.8607E-060.310 1.3679E-060.320 6.6608E-07...


In *n*_2_ datasets, the data are always presented numerically. An example can be found at data-n2/main/SiO2/Flom.yml. *n*_2_ is expressed in m^2^/W:


DATA:- type: tabulated n2data: |0.772 2.07e-201.030 2.23e-201.550 2.42e-20SPECS:n2_method: Z-scanpulse_duration: 280e-15 140e-15 97e-15


It is noteworthy that for *n*_2_, information on the measurement method and pulse duration is essential for comparing data from different sources. This information is specified in the optional SPECS field.

#### COMMENTS and SPECS

The COMMENTS field can incorporate additional information to provide context to the data, while the SPECS field presents structured data in key-value pairs, giving machine-interpretable insights into specific measurement conditions or additional properties.

In the initial example, the SPECS entries clarify that the refractive index is not absolute and is measured relative to air, that the wavelength is gauged under atmospheric conditions rather than in vacuum, and that the data is applicable at a temperature of 20 °C. In the preceding example concerning *n*_2_, the SPECS delineate the use of the Z-scan measurement method, specifying pulse durations of 280 fs, 140 fs, and 97 fs for the corresponding data points. Furthermore, the SPECS section is adaptable to encapsulate an enhanced depth of information regarding the material, as illustrated in the forthcoming example for SCHOTT N-BK7 glass:


SPECS:...thermal_dispersion:- type: “Schott formula”coefficients: 1.86e-06 1.31e-08 −1.37e-11 4.34e-07 6.27e-10 0.17nd: 1.5168Vd: 64.17glass_code: 517642.251glass_status: standard...acid_resistance: 1.0alkali_resistance: 2.3phosphate_resistance: 2.3


Inclusion of additional information on optical glasses makes the data records suitable for use in optical design software.

### Notes on data records

Adhering strictly to the syntactical rules of YAML is paramount. This adherence includes the mandatory use of spaces for indentation (tabs are prohibited) and the application of UTF-8 encoding without BOM, ensuring consistency and readability across diverse data records. While not a stringent requirement, a uniform format for similar entries, particularly references, is encouraged to optimize the user experience and enhance data interpretability.

Unless explicitly defined, non-prefixed SI units (e.g., watts, meters, seconds) are the default to ensure unambiguous and standardized data representation and interpretation. For instance, *n*_2_ is expressed in units of m^2^/W. However, an exception exists for the wavelength, which is always specified in micrometers to comply with a general practice accepted in optical design and, in particular, to allow the direct use of published dispersion formulas that traditionally assume wavelength expressed in micrometers.

## Technical Validation

Since the described dataset^5^ represents an extensive array of data compiled by the work of thousands of researchers over more than a century, we cannot verify the accuracy of every individual record. Instead, we rely on the peer-review process employed by publishers of scientific and technical journals, and on the experience and reputation of optical material manufacturers publishing their material properties. However, we make every effort to ensure the correctness of the data extraction and conversion process, as well as the consistency of the information included in the dataset. In particular, the following steps are typically involved in the process of adding a new data record.

1. Verification and documentation of data attribution: We trace the origin of the data to the first publication where it appeared and include the corresponding information in the data record file, ensuring users can validate the origins of the data. If the original data were re-analyzed, or a combination of data from multiple sources was used in a later publication (for example, to produce a dispersion formula valid in an extended wavelength range), this fact is also documented in the REFERENCES field of the data record file.
2. Verification of accuracy of data extraction: Several tests are used to ensure the absence of errors in the conversion process:
  - Manual number-by-number comparison: This test is routinely performed when the data is represented by a relatively small amount of numerical values, e.g., coefficients of a dispersion formula.
  - Plot comparison: We use Python scripts that automatically read YAML data record files and plot the included optical constants as a function of wavelength. Comparing these plots with those included in the original publications is a robust tool for spotting inconsistencies.
  - Looking for data abnormalities: A deviation of a data point from the general trend in the data record is usually an indication of a data extraction error. All such abnormalities are manually compared against the original data, and necessary corrections are made.
  - Cross-entry comparison: Plotting data entries for the same material from different sources on the same plot may help reveal a systematic error in a particular data record if a corresponding curve deviates from a general trend. An example of a Python-based user interface allowing for easy data comparison is given in the following section.
3. Testing of data file adherence to the YAML standard: This is typically performed by verifying the as-expected operation of several scripts used to access the data and relying on standardized YAML processing libraries. Error or warning messages generated by these scripts indicate a problem in the data record file that must be identified and corrected.
4. User feedback: This is our strongest defense line in assuring the integrity of the data records. The users report errors via the GitHub page of the *refractiveindex.info* project or by directly contacting the maintainer. Each report is analyzed, and necessary corrections are promptly implemented.

## Usage Notes

The described dataset^5^ is a collection of human-readable YAML files, meticulously organized for user convenience. While these files can be directly individually accessed for specific optical constants of materials, organizing them in a logically-structured way and employing computer programs for data retrieval and analysis unleashes the dataset’s comprehensive utility. The YAML format, a standardized data serialization language, ensures that the data files are easily parsable with libraries such as PyYAML for Python and libyaml for C/C++.

The *refractiveindex.info* database is build upon the dataset described in the previous sections and include additions aimed at simplifying the access and navigation through the data. The main addition is a descriptor defining a hierarchical structure akin to a library. In this setup, each data record is akin to a "page," housed within a "book," and all such books are systematically arranged on different "shelves." This logical structure is defined and maintained through YAML-based catalog files, that categorize each data record following the ‘library’ analogy and indicate the relative path of the corresponding data records.

The library is defined by two catalog files: catalog-nk.yml for linear optical properties and catalog-n2.yml for nonlinear properties. Below is an excerpt from the catalog-n2.yml file, exemplifying the integration of HTML typesetting and showcasing optional entries like ‘DIVIDER’ and ‘info’. The ‘DIVIDER’ is employed to separate distinct groups of books or pages clearly, while ‘info’ links to an HTML file furnishing extra details about a particular shelf, book, or page.


- SHELF: mainname: “MAIN - simple inorganic materials”content:- DIVIDER: “Al - Aluminates, Aluminium garnets”- BOOK: BeAl2O4name: “BeAl2O4 (Beryllium aluminate, chrysoberyl)”info: “main/BeAl2O4.html”content:- PAGE: Adairname: “Adair 1989”data: “main/BeAl2O4/Adair.yml”...- BOOK: MgAl2O4name: “MgAl2O4 (Magnesium aluminate, spinel)”info: “main/MgAl2O4.html”content:- PAGE: Flomname: “Flom et al. 2015”data: “main/MgAl2O4/Flom.yml”- PAGE: Adairname: “Adair et al. 1989”data: “main/MgAl2O4/Adair.yml”...


In this schema:

- ‘SHELF’ represents a specific category or collection of materials.
- ‘BOOK’ denotes a particular material.
- ‘PAGE’ entries detail individual data records associated with a material.
- ‘DIVIDER’ assists in visually and logically separating related materials or data records, enhancing the navigation experience.

The ‘info’ entry specifies paths to additional HTML-based information, enabling users to access in-depth insights. Each ‘PAGE’ entry is linked with a ‘data’ field, pointing to the exact location of the data record’s YAML file within the dataset. The ‘name’ entry provides a “long” name for a shelf, book, or page in HTML typesetting. Paths to the data files for the linear (*nk*) and nonlinear (*n*_2_) subsets of the database, as well as to the HTML files with additional information (*info*), are relative to the data-nk, data-n2, and info directories, respectively, all located in the database’s root directory.

It’s important to note that users can create alternative ‘catalog’ files tailored to their specific needs. For instance, a customized catalog can contain only a subset of the dataset that is relevant for a particular application or study.

**Fig. 1:**
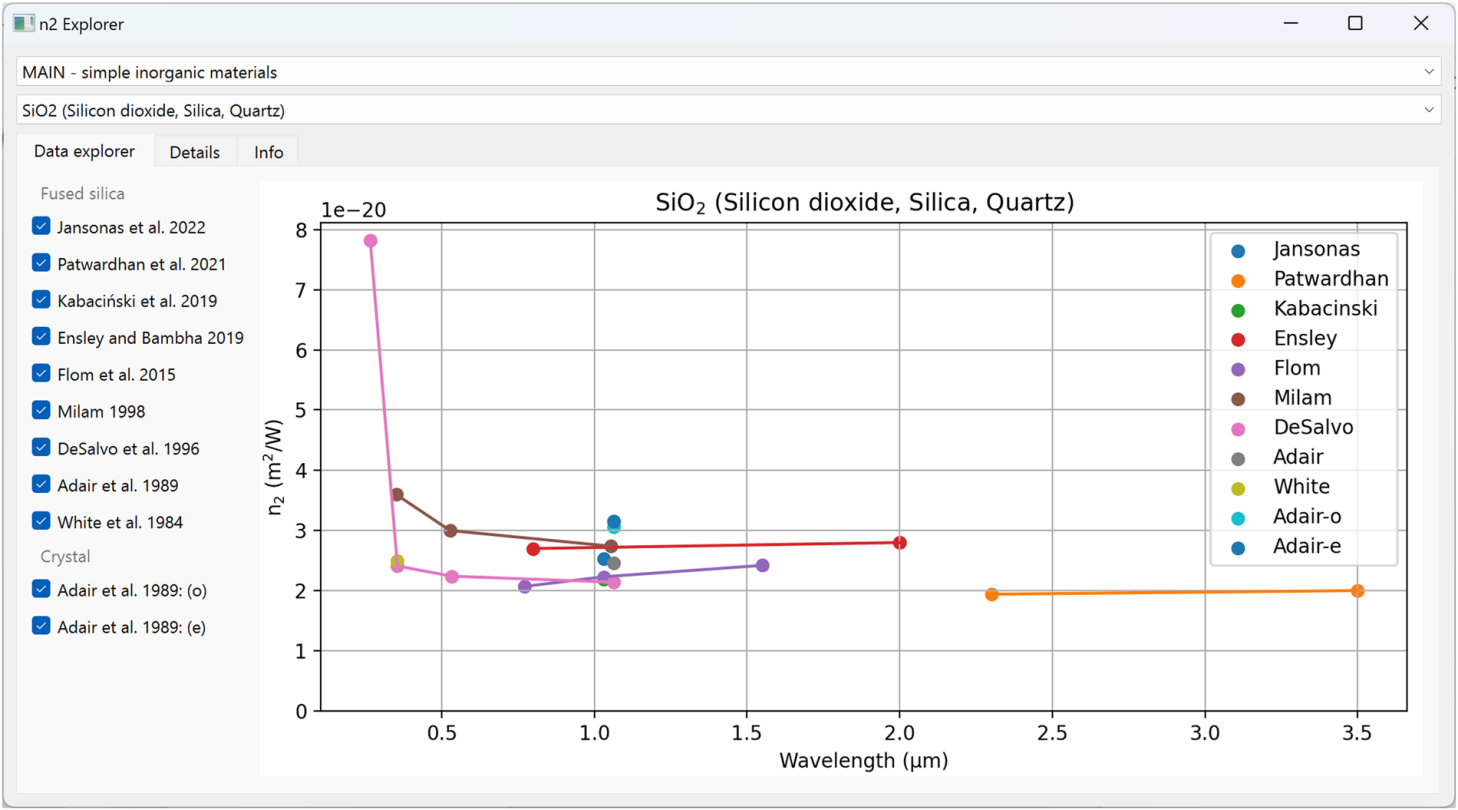
The n2explorer.py script’s graphical user interface facilitates visual navigation through the *n*_2_ data. The nkexplorer.py script provides a similar interface for exploring linear optical constants.

To aid users in navigating the data, two Python scripts, nkexplorer.py and n2explorer.py, are housed in the tools folder in the root directory of the *refractiveindex.info* database. These utilities, boasting a QT-based graphical interface, facilitate the location and comparison of data from a variety of sources. Figure 1 displays the n2explorer.py interface, illustrating the visual comparison of *n*_*2*_ data for SiO_2_ sourced from multiple publications.

Furthermore, users have the option to access the database through the RefractiveIndex.INFO website (https://refractiveindex.info). This online platform facilitates the browsing of data records and computations of various optical properties linked with *n* and *k* constants, such as Abbe numbers, reflectance, and Brewster’s angle.

A variety of third-party scripts and web applications that harness the *refractiveindex.info* dataset can be found, notably on GitHub. These tools offer users alternative avenues to efficiently access and employ the data.

## Data Availability

The dataset described here, which represents the core of the *refractiveindex.info* database, is available at *Figshare*^5^. It presently (as of December 2023) contains 3135 data records on 605 materials in the part of the dataset corresponding to linear optical properties (*nk*), and 193 records on 89 materials in the part corresponding to nonlinear optical properties (*n*_2_). The code that underpins the *refractiveindex.info* database is made accessible under the Creative Commons Zero (CC0) license (https://creativecommons.org/publicdomain/zero/1.0). This license facilitates the unrestricted use, distribution, and modification of the code, making it widely accessible for various applications. The entire codebase, including detailed documentation, is publicly available on the *refractiveindex.info-database* GitHub project (https://github.com/polyanskiy/refractiveindex.info-database). This repository is regularly updated, ensuring it evolves to meet the ongoing needs of both the scientific and engineering sectors. For additional utility, users can explore the *refractiveindex.info-scripts* project on GitHub (https://github.com/polyanskiy/refractiveindex.info-scripts), which offers scripts for deriving optical constants from established models and tools for converting Zemax glass catalogs to the dataset’s YAML format. It is essential to note that the data encapsulated within the *refractiveindex.info* dataset is meticulously curated from publicly available sources. This includes peer-reviewed journals, authoritative books, and manufacturer datasheets, ensuring that the dataset is not only expansive but also anchored in reliability and veracity. Each data record within the dataset explicitly cites the source, offering users a pathway to delve deeper into the original data and its context. All journal papers from which data are presently used in the *refractiveindex.info* dataset, excluding those without a DOI identifier, are included in the following reference list^6–471^. By integrating a comprehensive data collection, adopting a standard-based data file format, ensuring ongoing updates, and maintaining open access, the *refractiveindex.info* emerges as an essential tool for researchers, engineers, and students delving into the complex world of optical constants and material properties.
